# Variant profiles of genes mapping to chromosome 16q loss in Wilms tumors reveals link to cilia-related genes and pathways

**DOI:** 10.18632/genesandcancer.207

**Published:** 2020-10-06

**Authors:** Eiko Kitamura, John K. Cowell, Chang-Sheng Chang, Lesleyann Hawthorn

**Affiliations:** ^1^ Georgia Cancer Center, Augusta University, Augusta, GA, USA

**Keywords:** Wilms tumor, chromosome 16q, cilia-related genes, cilia pathways

## Abstract

Background: Wilms tumor is the most common pediatric renal tumor and the fourth most common malignancy in children. Chromosome 16q deletion(del) or loss of heterozygosity (LOH) has been correlated with recurrence and overall poor prognosis, such that patients with 16qLOH and 1p allelic loss are treated with more aggressive chemotherapeutic regimens.

Methods: In the present study, we have compared the variant profiles of Wilms tumors with and without 16q del/LOH using both data available from the TARGET database (42 samples) and tumors procured from our legacy collection (8 samples). Exome-Seq data was analyzed for tumor specific variants mapping to 16q. Whole exome analysis was also performed. An unbiased approach for somatic variant analysis was used to detect tumor-specific, somatic variants.

Results: Of the 72 genes mapping to 16q, 42% were cilia-related genes and 28% of these were found to carry somatic variants specific to those tumors with 16qdel/LOH. Whole exome analyses further revealed that 30% of cilia-related genes across the genome carried alterations in tumors both with and without 16qdel/LOH. Additional pathway analyses revealed that many cilia-related pathway members also carried deleterious variant in these tumors including Sonic Hedgehog (SHh), Wnt, and Notch signaling pathways.

Conclusions: The data suggest that cilia-related genes and pathways are compromised in Wilms tumors. The genes on chromosome 16q that carry deleterious variants in cilia-related genes may account for the more aggressive nature of tumors with 16q del/LOH.

## Introduction

Wilms tumor (WT) is the fourth most common pediatric cancer and affects approximately 1 in 10,000 children in Europe and North America. It typically presents as a complex embryonal tumor with triphasic histology (blastemic, epithelial and stromal components) and may also display cartilage, osteoid, and neural elements adding to the complexity of these tumors [1]. Although having a relatively good overall survival (>90%), due to a combination of surgery and more recently radiation/chemotherapy, there is also a subgroup of patients with poorer overall survival [2]. The stage at diagnosis is important to some extent in this determination, as is the histological subtype. WT show favorable (FHWT) or diffuse anaplastic (DAWT) histology, where the anaplastic histology is defined by the presence of atypical, polyploidy mitotic figures, large nuclei and hyperchromasia [3]. Bilateral tumors, usually associated with hereditary forms of the disease, cannot be treated with bilateral resection and therefore need alternative therapeutic strategies [4]. There is no apparent correlation between histology subtype and tumor stage, and only 50% of children that suffer relapse will survive. In addition, there is a high incidence of late radiation morbidity in patients undergoing adjuvant radiotherapy for Wilms tumor, significant adverse events and treatment-related risk factors in long-term Wilms tumor survivors and a high risk of second malignant neoplasms, presumed to be due to treatment. 

Studies aimed at defining the molecular characteristics of relapsing WTs have identified abnormalities associated with poor outcomes including loss of heterozygosity (LOH) at 16q [5, 6].

These observations have been confirmed by several groups in large studies in the National Wilms’ Tumor Study Group (NWTSG) study groups 3 and 4 as well as the United Kingdom Children’s Cancer Study Group (UKCCSG) studies [6, 7]. In the prospective NWTSG5 study, involving >2000 samples, 16% showed 16q LOH, and a significant correlation was found between poor prognosis and relapse within 2 years of treatment [5]. Additionally, LOH at 16q has been associated with a 2.7-fold increased risk of death in favorable histology tumors [6]. Moreover, a recent meta-analysis involving 10 studies and 3385 patients concluded that LOH at 16q was significantly associated with WT relapse in both histological subtypes [8]. These studies now clearly demonstrate that there is a subset of WT that are dependent on genetic events on 16q which determine poor outcome. Chromosome 16q and/or 1p allelic loss status are used to classify patients within the NWTSG therapeutic protocol to receive more rigorous chemotherapies [3, 5].

Knudson’s landmark ‘two hit hypothesis’ [9] provided proof-of-principle that tumor suppressor genes could be identified by examining genes mapping to single allele deletions accompanied by variants in second alleles at these loci. However, attempts to identify loci at chromosome 16q have largely been futile due to the extensive regions of LOH typically observed, possibly suggesting that several genes may act in concert to contribute to the more aggressive phenotype.

We have used data generated through the Therapeutically Applicable Research to Generate Effective Treatments (TARGET) initiative, which has enabled comprehensive characterization of high-risk Wilms tumor cases, defined as having either favorable histology (FHWT) that relapsed or diffuse anaplasia (DAWT). These samples were combined with a legacy set of samples to define variant-containing genes on chromosome 16 which may contribute to Wilms’ tumorigenesis or the more aggressive nature of tumors with 16q del/LOH. As a result, it became apparent that a large percentage of the tumor-specific variants detected in WT with 16q loss and/or LOH affected genes that encoded cilia- related proteins. A more extensive genome analysis beyond chromosome 16 demonstrated tumor-specific, variants in additional genes associated with ciliogenesis.

## RESULTS

### Microarray analysis of copy number and LOH

**Figure 1:**
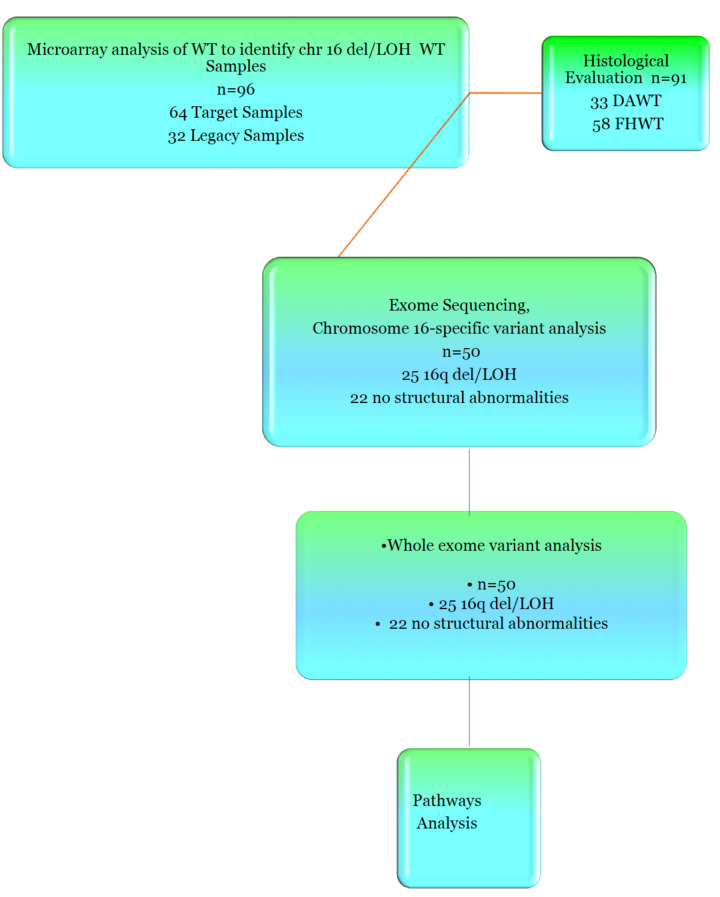
Flow Chart of Experiments. Experiments began with the use of SNP microarrays to identify samples with chromosome 16qdel/LOH. We analyzed these samples for histological correlates. Exome sequencing was then performed, and variants located on chromosome 16q were analyzed. Whole genome exome analysis was then carried out, followed by pathways analysis.

**Figure 2:**
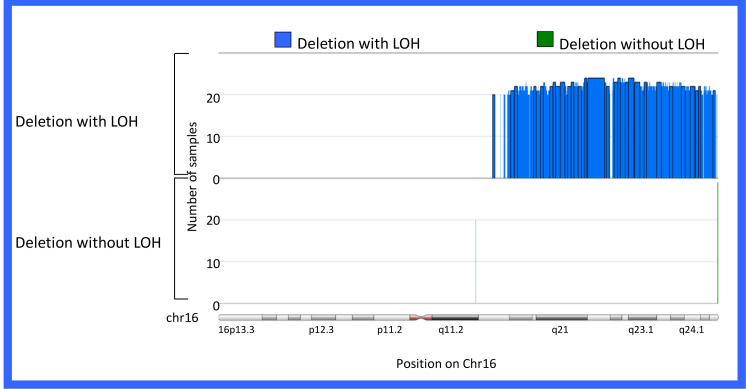
Regions of Loss/LOH on Chr16q. Depiction of chromosome 16 showing regions where commonly changed in more than 20 samples. The Y-axis represents number of samples harboring deletion with LOH or deletion without LOH and the x-axis shows position on chr16. Deletion with accompanying LOH was observed in 24/69 Wilms tumor samples (blue). Also shown are regions of deletion without LOH in 8/69 samples (green). Overall, 46% of samples carried structural abnormalities on Chr16.

(Figure 1) shows the experimental layout in order to clarify the number and types of samles used in each of the experiments. In our previous study [10] we defined three types of 16q abnormality; deletion with accompanying LOH, LOH without 16q deletion and copy number abnormalities (CNA) without LOH, presumably resulting from chromosome loss from a tetraploid cell. Of 69 paired samples from the TARGET project, 32 (46%) carried either deletions or LOH involving 16q, which included 24 samples with deletion associated with a LOH (35%) and 8 samples (11%) carrying deletions without accompanying LOH (Figure 2).

Deletions with accompanying LOH extended the entire length of 16q, while deletions without accompanying LOH, localized to chr16q11.2 (46506039-46539392) and 16q24.3 (90158838-90287536). These tumors are now collectively termed 16qdel/LOH samples. Combining the TARGET data with that previously reported (Hawthorn and Cowell 2011), 96 samples were available for analysis (Supplemental Table1).

**Figure 3:**
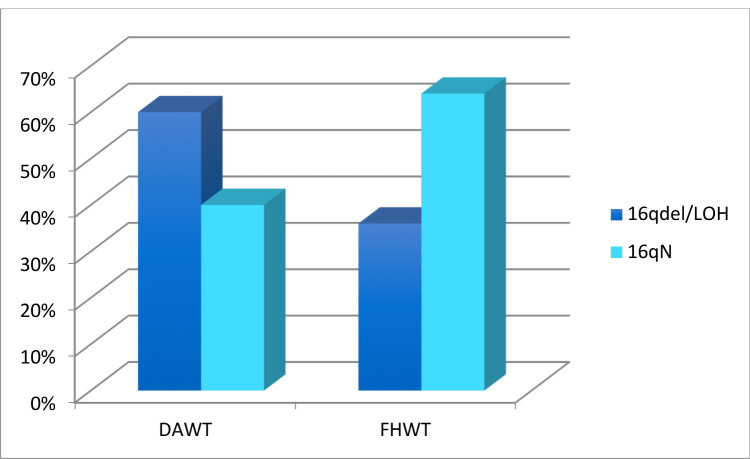
Histology as a Function of Chromosome 16q Status. Graph comparing WT samples between 16qdel/LOH and 16qN in DAWT or FHWT histological subtypes. The y-axis indicates percentage of samples with16qdel/LOH and 16qN observed in each histological subtype. In the DAWT group, 60 % of total samples (20/33) were 16qdel/LOH and 40 % (13/33) were16qN. On the other hand, 36 % of FHWT samples (21/58) were 16qdel/LOH while 64 % (37/58) were 16qN.

Within this series of tumors, both histological evaluation and assessment of chromosome 16q status was available from 91 cases, consisting of 33 with DAWT and 58 with FHWT. 60% (20/33) of tumors with DAWT histology and 36% (21/58) with the FHWT histological subtype had loss/deletion of chromosome 16q . Figure 3 shows that structural abnormalities of 16q are significantly associated with the DAWT histology (Welch 2-sided *t*-test *p* = 0.02).

**Table 1:** Genes Mapping to Chromosome 16q Frequently Altered genes in WT. Shown are the 20 top genes of a total 91 carrying one or more homozygous somatic variants that are predicted to be damaging and/or deleterious at the protein level.

Gene Symbol	# Samples in all 50 Samples	# Samples in 25 16qdel/LOH Samples	# Samples in 22 16qN Samples
HYDIN	8	7	1
NQO1	8	8	0
DRC7	7	7	0
ADAMTS18	6	5	1
CDH3	6	6	0
KCNG4	6	6	0
ZNF778	6	6	0
CTU2	5	4	1
MT1A	5	5	0
PKD1L2	5	4	1
PMFBP1	5	4	1
TAF1C	5	4	1
ZNF19	5	5	0
ABCC12	4	4	0
ACSF3	4	4	0
BCMO1	4	4	0
CDH11	4	4	0
CNGB1	4	4	0
PHLPP2	4	4	0
ZCCHC14	4	3	1

### Variants detected in WT

Exome-Seq data from 50 WT samples was analyzed, 42 samples from the TARGET project and 8 samples from the legacy collection. The annotated variants were filtered for predicted functional effects as outlined in the Materials and Methods section and categorized into missense variants, frameshifts, deletions/insertions and stop gain/losses. A total of 24,271 heterozygous-/homozygous- somatic variants predicted as Damaging and/or Deleterious were identified in 10,789 genes. The full list of somatic variants is available in Supplemental TableS2. It should be noted that the number of variants are higher than other published works due to the fact that we did not filter out variants that were listed in dbSNP but instead focused on variants that were present in the tumors and not in the matched normal samples, ie “tumor-specific/somatic”.

**Figure 4:**
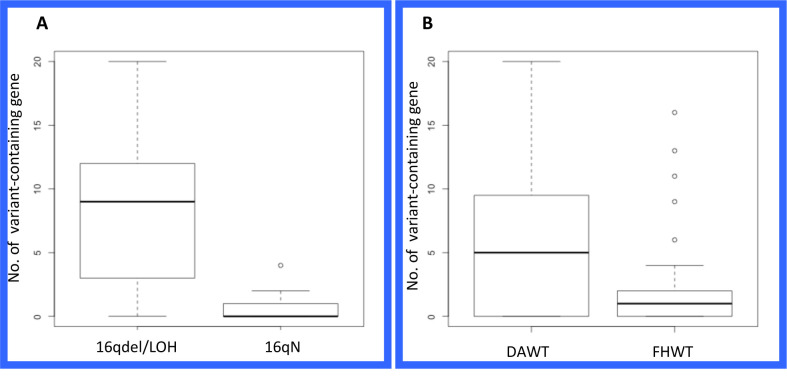
Box Plots of Sample Characteristics and Somatic Variants at Chromosome 16q. A. The box plot displays16qdel/LOH group having a median of 8.1 (homozygous, deleterious/damaging) variant-containing genes/patient mapping to chromosome 16q and the 16qN group having a median of 0.59 variant-containing genes/patient. The difference between the two groups is significant at *p* = 2.9e-07 using a Welch two-sample t-test. B. Box plot shows that the median number of variant-containing genes is more prevalent in DAWT histological class (5.8 /patient) than on the FHWT class (2.7 /patient). The difference is significant at *p* = 0.07 using a Welch two-sample t-test.

To examine the variant profiles in the 16qdel/LOH samples, exome data from 50 samples were analyzed, 25 of which were16qdel/LOH and 22 with no structural abnormalities of 16q and three that had no accompanying copy number data available. Using the pipeline described in the Materials and Methods section, we identified 131 homozygous or hemizygous in the samples with allelic loss, somatic variants in 91 genes that mapped to 16q in the 16qdel/LOH samples. Chromosome 16N tumors carried somatic variants in 13 genes, 10 of which overlapped with the variants in the 16qdel/LOH samples and 3 genes that were unique. The 16qdel/LOH group carried a median of 8.1 (homozygous or hemizygous in the samples with allelic loss, deleterious/damaging) variant-containing genes mapping to chromosome 16q per patient, while 16qN group carried a median of 0.59 genes per patient (See Figure 4). The top 20 variant-containing genes are shown in Table 1. The complete list of all somatic variants mapping to 16q in tumors with and without 16q abnormalities is available in Supplemental TableS3.

As envisaged, the number of variants-containing genes mapping to chromosome 16q is higher in the DAWT group. Figure 4b shows that there is also a statistically significant difference (*p* = 0.07) between the number of variant-containing genes carried by the patients with DAWT (median= 5.8 genes/patient) compared with patients with FHWT (median=2.7 genes/patient). There are 33 genes overlapping between the 2 histological subtypes, 31 uniquely altered in the DAWT and 18 unique to the FHWT subtypes.

**Figure 5:**
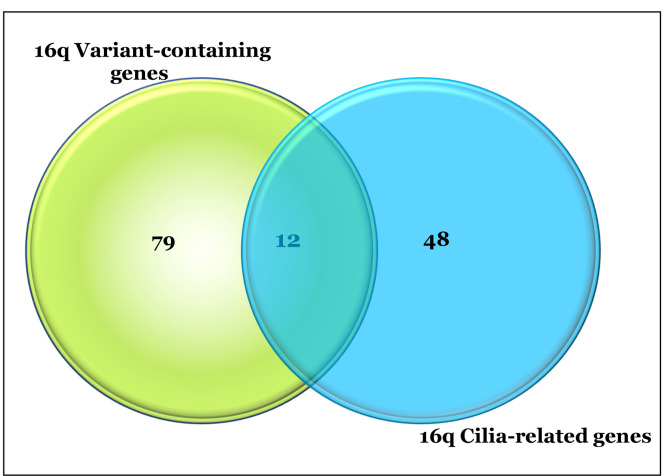
Venn Diagram of Cilia-related Genes Mapping to Chr 16q. The diagram shows the overlap of 60 Cilia-related genes mapping to chromosome 16q (in red) and the 91 variant-containing genes detected using our analysis (in blue). The overlap between the two groups is 12 genes.

Analysis of gene classification using GO Genesets in GSEA revealed that GO-Cilium was significantly (*q* = 3.5^e-2^) enriched in the 91 variant-containing genes mapping to 16q. The CilDB was then queried using chromosome location and 60 genes were identified that mapped to chr16q. The 91chromosome 16q variant-containing genes were then cross-referenced with those in the CilDB which identified 12 that overlapped (see Figure 5). Of note, however, many of these genes are included in the CilDB as a result of genome-wide transcript expression analyses and have not been fully validated as having cilia-related functionality. We, therefore, further curated genes that had also been reported in the literature as ‘cilia-related’, by GO functional categorization and the DAVID functional annotation tool.

Table 2 shows 12 of the cilia-related genes which contain somatic variants. Notably, 17/25 16qdel/LOH samples carry variants in cilia-related genes mapping to 16q. It is noteworthy that only one patient without a 16q abnormality carried homozygous somatic variants in any of these genes. 

### Whole exome analysis of WT

Given the overwhelming number of cilia-related genes on chromosome 16q that carried somatic variants, we extended the variant profiles of cilia-related genes to the entire exome and also determined whether there was a difference in variant profiles between tumors with (*n* = 25) and without (*n* = 22) 16q structural abnormalities. 23,429 variants were detected in the 47 tumor samples that were predicted to be detrimental at the protein level as outlined in the Materials and Methods. A total of 4960 and 4679 genes respectively carried somatic variants in two or more samples with and without 16q abnormalities and 3577 genes overlapped between the two groups. Of these, 1383 and 1102 genes respectively were unique to the tumors with and without 16q abnormalities. Table 3 shows the 30 most frequently altered genes for each of these groups. The complete list of somatic variants is available in Supplemental Table2.

**Figure 6:**
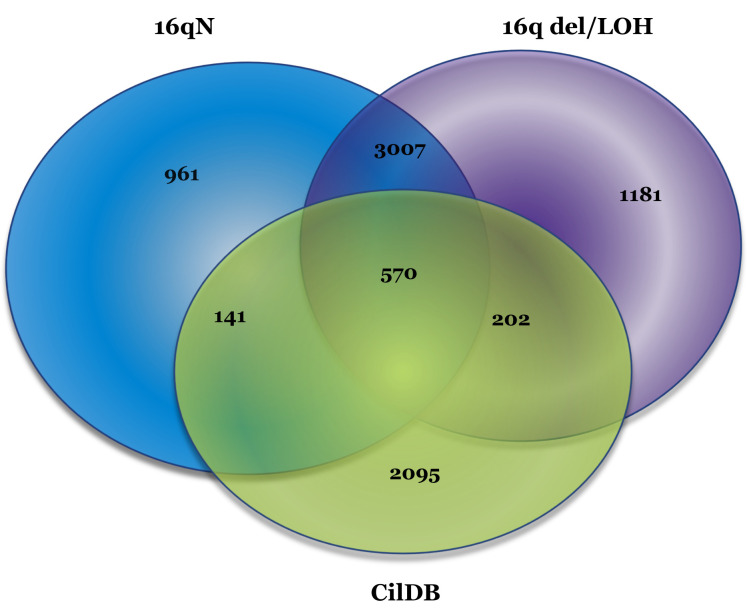
Venn Diagram of Variant-containing Genes in CilDB. Genes that are listed in the CilDB (3008 human genes) are shown in green, the 16qN samples (4679 genes) in blue and the 16qldel/LOH samples (4960 genes) shown in red. The number of genes overlapping between 16qdel/LOH samples and the CiliaDB is higher than the number overlapping between the 16qN samples and the CiliaDB. The gene lists for each of these categories is available in Supplemental Table4A.

Analysis using the CilDB showed a large number of genes overlapping with cilia-related functions from tumors with and without 16q abnormalities and these findings are shown in Figure 6, where it can be seen that 570 genes in both sample types overlap with CilDB genes, 141 are unique samples with no 16q abnormalities and 202 uniquely overlap between the 16qdel/LOH and the CilDB. The genes that comprise each of the overlaps are defined in Supplemental TableS4A.

Interestingly, genes that carried variants in the 16qdel/LOH samples were identified by this analysis as having deleterious variants in tumors with and without 16q abnormalities. For example, the *HYDIN* gene had homozygous, somatic variants in 7/25 16qdel/LOH samples but only 1/22 homozygous variants were detected in the 16qN samples (Table 2). Using whole exome analysis, however, 12/25 and 11/22 somatic variants were detected in the two groups respectively, however not all of these were homozygous (or hemizygous in the samples with allelic loss) variants (Supplemental Table4B).

The 570 variant-containing genes identified in the CilDB and overlapping between tumors with and without 16q abnormalities (Figure 6) were also analyzed using DAVID and GSEA to refine the list of cilia- related genes. Using the functional annotation clustering algorithm in DAVID the most highly enriched annotation cluster included cilia biogenesis, cilium assembly and cilium morphogenesis with an enrichment score of 14.04. Other annotation clusters included with high enrichment scores included cilium movement/primary ciliary dyskinesia (ER=7.7), dynein heavy chain/ axonemal dynein complex (ER=7.47) and intraciliary transport/primary cilium (ER=3.1). Using GSEA on the same 570 overlapping genes (with an FDR q value of <0.05), 266 genes were identified as belonging to the GO classifications of GO_ CILIUM , GO_ CILIUM MORHOGENESIS, GO_ CILIUM ORGANIZATION, GO_ CILIARY PART, GO_ CILIARY PLASMA, GO_ PRIMARY CILIUM, GO_ MEMBRANE, GO_ AXONEME, GO_ AXONEME ASSEMBLY, GO_ CENTROSOME, GO_ CENTRIOLE. One hundred and forty-three of the 266 carried variants in at least 25% of 16qdel/LOH samples and 149 in 25% of the tumors without 16q abnormalities.

**Table 2:**
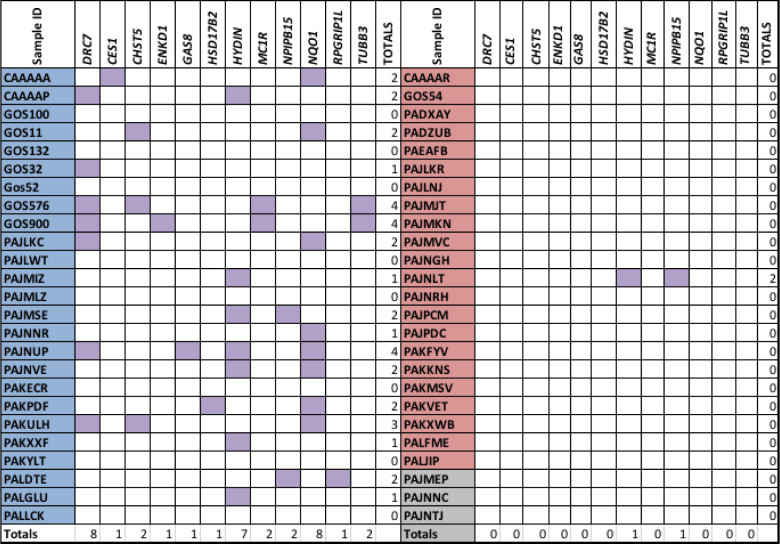
List of Cilia-Related Genes Mapping to 16q. List of cilia-related genes mapping to 16q and carrying somatic variants in the 50 listed samples. 16qdel/LOH samples are shaded in blue while 16qN samples are shaded in red. The samples shaded in grey have no copy number information available. The purple- shaded boxes denote those samples that have homozygous or hemizygous in LOH samples that are deleterious/damaging variants in the genes listed along the top. It is notable that the vast majority of these occur in 16qdel/LOH samples and only 1 16qNsample has variants in any of these genes.

### Established cilia-dependent pathways

**Figure 7:**
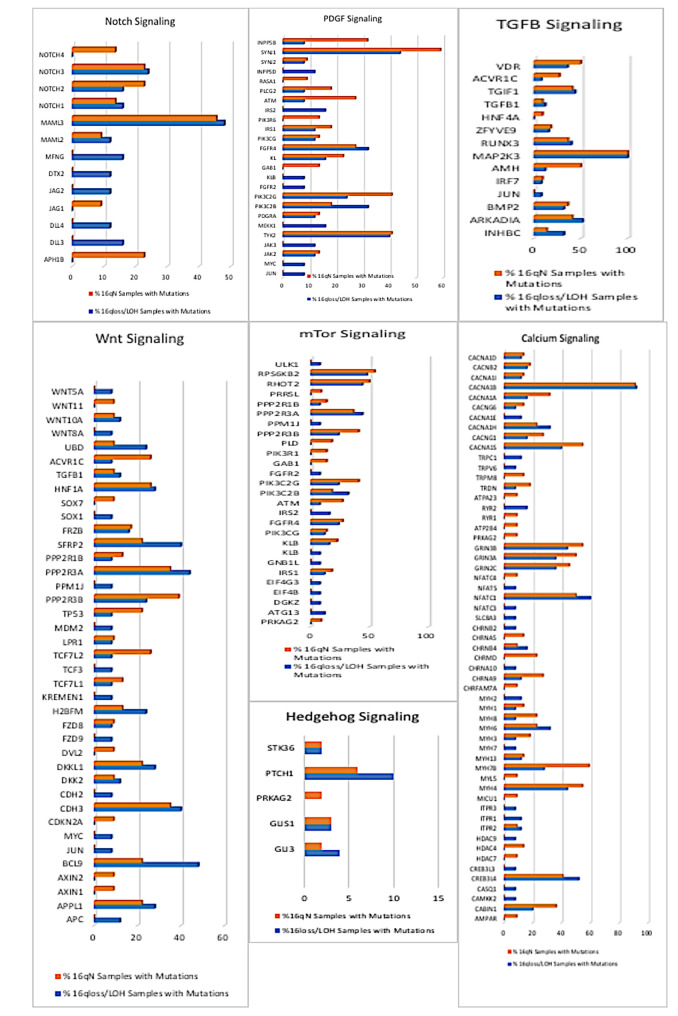
Deleterious Variants in Cilia-Related Pathways. The X-axis of each bar chart shows the names of the genes in each pathway that carry mutations. The y axes show the number of mutations in each of the genes. The orange bars represent the 16q- samples and the blue bars represent the number of mutations in the 16q+ samples.

We next examined well-established, cilia-dependent pathways including Sonic Hedgehog (SHh), Wnt, Platelet-derived growth factor receptor (PDGFR), Notch, TGF-B and mTOR for variants in their component molecules. The pathway members and the percentage of samples with variants in tumors with and without 16q abnormalities are shown in Figure 7.

Although there is a large degree of overlap in the variants for each pathway, some of the genes in each of these pathways differ. For example, in the Wnt Signaling pathway APC, BCL9, KREMEN, c-JUN, FDZ9 and MDM2 variants are evident in the 16qdel/LOH samples exclusively and conversely, AXIN, CDKN2A, SOX7 and WNT11 are altered solely in the tumors without 16q abnormalities. The SHh pathway shows larger percentages of somatic variants in the PTCH1 and GLI3 genes in the 16qdel/LOH samples, while PIK3R1, ATM and PLD genes are more frequently altered in tumors without 16q abnormalities. All pathways are shown in Supplemental Image1 (SI1).Overall, a down-regulation of these pathways due to inactivating somatic variants is predicted.

### Variant containing genes previously reported in Wilms tumor

Genes that have been previously reported to carry variants in Wilms tumors also were found to carry variants in this study, however using our analysis pipeline these were not determined to be frequent events. For example, in AMER1, we detected somatic variants in 6% of samples, CTNNB1 (0%), DICER1 (4%), DGCR8 (6%), DROSHA (10%), MLLT1 (16%), SIX2 (2%) and TP53 (24%). The list of the most frequently reported WT-associated variant-containing genes is shown in Table 4. 

**Table 3:** List of Frequently Altered Genes. Genes most frequently altered across the genome in WT samples for both 16qdel/LOH and 16qN tumors. Genes were identified using Exome-seq and the analysis pipeline described in Materials and Methods. The most frequently altered genes overlap substantially between the two groups.

Gene Symbol	%16qdel/LOH Samples with Somatic variants	Gene Symbol	%16qN Samples with Somatic variants
GPRIN2	100	GPRIN2	100
KCNJ12	100	KCNJ12	100
MAP2K3	100	MAP2K3	100
OBSCN	100	OR4C3	100
OR4C3	100	PDE4DIP	100
PDE4DIP	100	BCLAF1	95.5
BCLAF1	96	DNAH5	95.5
GPR98	96	KRT32	95.5
NEB	96	MUC3A	95.5
NRAP	96	NEB	95.5
CACNA1B	92	SCGB1C1	95.5
RHBG	92	ANKRD36	90.9
ACAN	88	CACNA1B	90.9
COL4A3	88	CMYA5	90.9
DNAH11	88	MKI67	90.9
FCGBP	88	OBSCN	90.9
MKI67	88	PLIN4	90.9
MUC20	88	PRIM2	90.9
PRIM2	88	RNF43	90.9
PTCHD3	88	RP1L1	90.9
SCGB1C1	88	KRT37	86.4
ALPK2	84	MUC20	86.4
CMYA5	84	NPIPB15	86.4
KRT32	84	NRAP	86.4
OR2B11	84	PCNT	86.4
OR9G1	84	PTPRH	86.4
PCNT	84	PZP	86.4
ALDH1B1	80	ACAN	81.8
APOB	80	BPIFB4	81.8
CA6	80	DRC7	81.8

## DISCUSSION

Loss of heterozygosity involving distinct regions of chromosome 16q been extensively correlated with poor outcome in Wilms’ tumors and is now included as a test in evaluating therapy options for WT patients. Although the incidence of 16q loss in WT tends to be 15-20%, [1, 10-14] the present study a much higher frequency (49%) was observed, most likely due to the TARGET samples being preselected as high-risk, i.e. having either favorable histology (FHWT) that relapsed or DAWT. Wittman et al [15] also reported a higher incidence of 16q allele loss in mixed-type as well as in diffuse anaplastic tumors, whereas epithelial and stromal tumors rarely exhibited 16q losses. It was also notable that chromosome 16 loss/ LOH was a less frequent event in the FHWT class and the majority were classified as DAWT and the number of variant–containing genes mapping to chromosome 16q was higher in the DAWT as opposed to the FHWT histologically classified samples. It is interesting that Gadd et al. [13] reported a higher percentage of overall variants across the genome in DAWT samples using the TARGET data.

The identification of genes mapping to chromosome 16q that play a role in WT tumorigenesis have been largely ineffectual and has led to the assumption that more than one gene mapping to this region could attribute to the more aggressive nature of tumors carrying 16q deletion or LOH. The observation of LOH is generally taken as being a mechanism of exposing recessive variants in gene critical to tumorigenesis and through our variant profiles focusing on 16q we found a remarkably consistent incidence of somatic variants in genes related to cilia structure and function. WT is considered an embryonal tumor arrested in early stages of kidney development. An early stage of this process involves the two-way induction of differentiation induced by the contact of the ureteric bud with the metanephric mesenchyme generating waves of differentiation signals as the bud invades the blastemal mass. During this process the kidney goes through differentiation into primitive stages of a pro-nephros and a meso-nephros which are normally degraded to give rise to the metanephric kidney [16]. Importantly, cilia have been shown to play an important role in early stages of this process [17] and ciliary structures can be seen in Wilms tumors reflecting their early stage in developmental arrest [18]. The frequent involvement of somatic variants in genes that are related to cilia, therefore, potentially represents a mechanism of sustaining embryonic status of cells in WT.

The large number of cilia-related genes both mapping to chr16q and containing variants in the 16qdel/LOH samples aroused our interest in the role of cilia in WT. Specifically we focused on 12 genes that were highly referenced in the literature. Out of 25 16qdel/LOH samples, only 8 did not have variants in cilia-related genes. Recent studies have shown that hundreds of proteins reside permanently or transiently in cilia. In the kidney, immotile or primary cilia are present on the apical (urinary /luminal) surface of epithelial cells from all tubular segments and are critical sensory and signaling centers. Primary cilia are solitary and immotile cellular appendages that serve as signaling hubs for many signaling pathways during development (see below). Defects in their structure/function result in a spectrum of clinical and developmental pathologies.

**Table 4:** List of Genes with Reported Mutations in Wilms Tumors. The table lists genes that have previously reported to carry mutations in Wilms tumors. The sample number heading refers to the number of samples with somatic variants detected in the gene out of a possible 50 total. The percentage heading is the percentage of 50 samples with the somatic variants. Starred genes refer to the genes reported by Gadd et al using the Target Database samples.

Gene	Sample #	% in 50 samples
AMER1	3	6.0
CTNNB1	0	0.0
DGCR8	3	6.0
DICER1	2	4.0
DROSHA	5	10.0
MLLT1	8	16.0
MYCN	0	0.0
SIX1	1	2.0
SIX2	1	2.0
TP53	12	24.0
WT1	2	4.0
XPO5	3	6.0
ARID1A*	1	2.0
ASXL1*	4	8.0
BCOR*	0	0.0
BCORL1*	0	0.0
COL6A3*	0	0.0
MAP3K4*	5	10.0
MAX*	0	0.0
NONO*	0	0.0

* genes reported by Gadd et al. 2017

Some of the genes that were detected through our analysis are related to motile cilia components and would not impact the primary or non-motile renal cilium, however it should be kept in mind that these tumors arise in the developing kidney and are blastemal in nature. Furthermore, the role of motile cilia in nephrogenesis has not been elucidated, for example, multi-ciliated cells, or cells that contain multiple motile cilia have been reported in fetal kidney tubules [19] and sporadically in an assortment of renal diseases [20, 21]. Additionally, despite the distinction between primary and motile cilia, it has been reported that there is a clinical overlap between Primary Ciliary Dyskinesia (PCD) and many of the non-motile-ciliopathies [22]. 

The most frequently altered gene detected in the present study was DRC7 (dyenin regulatory complex 7) a misnomer as this is a conserved ciliary protein localized to the outer microtubule doublets of the axoneme and is not associated with the dynein arms [23]. The role of this protein localizing to the axoneme implies that it may also be involved in primary cilia function, and play a role in nephrogenesis.

Several of the detected somatic variants mapping to 16q are involved in PCD a rare genetic disease that is inherited as an autosomal-recessive trait. For example, *HYDIN* was frequently found to carry variants in patients with chromosome 16q del/LOH. This gene encodes a protein that is integral to the central pair apparatus of motile cilia. Raidt et al [24] examined ciliary beat patterns for PCD patients carrying different somatic variants and reported that many of the cilia in nasal brushings of *HYDIN* mutation carriers were actually primary/immotile cilia, suggesting that the classification of these two subtypes of cilia is not definitive.

Another frequently altered gene was *NQO1*, a gene which has been reported as down-regulated in PCD biopsies of lung [25] and reduced expression leads to increased kidney injury in response to cisplatin [26].

*GAS8*, another PCD-related gene, encodes a subunit of the nexin-dynein regulatory complex and connects microtubule doublets. *GAS8* variants are associated with axonemal disorganization sometimes characterized by a partial loss of inner dynein arms [27, 28]. Interestingly, although *GAS8* localized to the microtubule axoneme of motile cilia it also localized to the base of non-motile/primary cilia [29] and the role in non-motile cilia has not been elucidated. Intriguingly, *GAS8* plays a role in the SHh signaling pathway. Evron et al [30] have shown in a murine model that Gas8 binds to Smoothened (*Smo*) and acts at the base of primary cilia as a regulator of *Smo* entry into the cilium following SHh pathway activation. In the absence of Gas8, *Smo* accumulation in the cilium is abrogated and that it cannot activate the *Gli* transcription factors that ultimately govern the expression of downstream genes.

The transition zone (TZ) is the proximal-most domain of the ciliary axoneme, found immediately distal to the basal body and is critical to cilium formation and functions as a portal that maintains the correct composition of the ciliary organelle. Variants in genes that affect TZ function result in wide range of ciliopathies. TMEM231 is mandatory for the localization of a subset of the MKS complex components to the TZ and to maintain ciliary protein composition. Another TZ protein, *RPGRIP1L* is also mutated in Merkel Syndrome (MKS) which is characterized by kidney cysts. Furthermore, variants in this gene cause Nephronophthisis (NPHP) which also characterized by kidney cysts. Genetic disruption of the transition zone disorders the ciliary localization of membrane-associated proteins including SHh-related *SMO* that requires the transition-zone proteins including *TMEM231* to accumulate within the ciliary membrane. Consequently, loss of any of these proteins leads to SHh-associated developmental defects [31].

Whole exome analysis also revealed that a large percentage of genes associated with ciliogenesis and mitosis carried somatic variants in both 16qN and 16qdel/LOH samples. Given that WT is a developmental tumor, these findings are notable. Although not reported specifically for Wilms tumor, an ever-increasing number of papers report on a decrease, loss, or distortion of the primary cilium in a variety of cancer types. It is commonly assumed that the cilium act as to control cellular proliferation by employing the same structural components required for chromosome segregation [32]. Loss of the cilium in cancer cells may, therefore, result in loss of these components and contribute to distorted cellular signaling [33]. Additionally, given the critical role of cilium in cell division, defects in cilia- related genes have become a viable explanation for ciliopathy phenotypes that appear during development as many cells are actively proliferating during this phase and therefore do not have cilia [34].

Further analysis of the established cilia-related pathways revealed that a large number of genes in these pathways carried somatic variants in the WT samples. The SHh was discussed above in the context of 16qdel/LOH homozygous variants detected in the *GAS8* and *TMEM231* genes. The SHh signaling plays an essential role in many aspects of embryonic development and tumorigenesis. The cilium functions as the transduction hub for SHh signaling [35]. Our analysis found 34% of samples carried *PTCH1* somatic variants and somatic variants of *GLI3*, *GLIS1* *STK3* and *PRKAG2* were also detected. Variants in *PTCH1* are associated with rhabdomyosarcoma [36], other studies report increased expression of *PTCH1* in pediatric solid tumors including Wilms tumor [37].

Many parallel and divergent lines of evidence point to Wnt Signaling (both canonical and non-canonical) as central pathways in nephrogenesis. The Wnt/β-catenin pathway is one of the multiple signaling pathways that cooperate in the initiation and progression of mesenchymal-epithelial transition and several members of the Wnt family have been implicated in the induction of epithelial renal vesicles (See 38 review). Wilms tumor development is tightly linked to nephron genesis and are frequently found within nephrogenic nests that resemble embryonic structures suggesting a block in the nephrogenic process by variants in the Wnt Signaling members. We report a large number of genes involved Wnt Signaling are altered in the WT cohort examined. The cilia and basal body function as regulatory mechanisms to govern Wnt signaling and signaling is mediated in the primary cilia. Additionally, ciliary-related proteins have been also shown to regulate Wnt signaling pathways.

Notch signaling also plays an elemental role in mammalian nephrogenesis. The differentiation of nephron progenitors requires the down-regulation of SIX2 and this accomplished by the Notch Signaling pathway [39]. Notch also regulates the process of nephron segmentation involving the differentiation of progenitor cells into the renal corpuscle, proximal tubule, loop of Henle and distal tubule [40]. Cells lacking the Notch signaling pathway fail to form these structures reminiscent of the disorganized nephrons typically observed in WT. The Notch signaling pathway plays a central role in left right symmetry and cilium length control. Our analysis revealed somatic variants in all 4 Notch receptors, with 23% of samples carrying somatic variants in *NOTCH3*.

The plethora of events, both in normal and disease states, involving calcium signaling is overwhelming, however studies of the role of calcium in kidney development has historically been largely overlooked. *PKD1* and *PKD2* encode proteins that interact to form a calcium permeable channel in response to mechano-sensory stimuli in ciliary membrane and this channel becomes dysfunctional due to variants in PKD patients. Other interacting members of *PKD2*, *TRPC1* and *TRPV4* show strong expression patterns in the embryonic proximal tubules and ureteric bud and genes carried somatic variants in our cohort. Additionally, calcium entry in response to extracellular stimuli results in calcineurin (*PPP3CA*) activation, and signal transduction from the cytoplasm into the nucleus through dephosphorylation and nuclear translocation of the transcription factor nuclear factor of activated T cells (*NFAT*). This initiates a cascade of transcriptional events involved in physiological and developmental processes [41]. We detected a large number of variants in members of the *NFAT* family with 55% of patients carrying somatic variants in *NFAT1*. Notably, *CABIN1*, a calcineurin binding protein which results in decreased *PPPC3A* expression is highly expressed in mesenchymal progenitor cells at the onset of metanephric kidney development has been found to be over-expressed in WT [42]. In the present study, we detected *CABIN1* somatic variants in 28% of samples assayed. 

Other established cilia-related pathways are less documented in terms of nephrogenesis or WT. Notwithstanding, the mTOR signaling pathway is mediated by the primary cilia and inappropriately activated in cyst-lining epithelial cells in human *ADPKD* patients and mouse models [43]. Defects in the autophagy pathway have been detected in PKD and ciliopathies display impaired autophagy [44]. TGFβ and PDGF signaling are intricately involved in the development of renal fibrosis [45].

Whole exome analysis concurred with similar studies using the target dataset with some major differences. The most frequently altered genes in our study did not correspond to those reported by Gadd et al [13]. This was primarily due to differences in data filtering for instance, their discovery set was limited to variants reported in COSMIC including nonsense, and frameshift variants and verified somatic missense and in-frame variants predicted to be damaging and not identified in 1000 Genomes series 3. We did not filter our data using these criteria but relied on detecting somatic variants that were tumor specific. As a result, our somatic variant list is much more extensive and we detected many genes that were altered in a large percentage of samples, however our sample size is smaller. 

*TP53* was the most commonly mutated gene in a study reported by GADD et al [13] using their discovery set analysis, the mutation rate was 22% and using our analysis, we calculated the variant-containing rate as 26% (Table 4). Similarly, we found our variant-containing rates concurred with those reported by Gadd et al for most of the WT-related genes except for *CTNNB1*, a gene reported by Gadd et al, as the second most frequently variant-containing gene whereas we detected no variants using our analysis pipeline. Presumably, the predictions for the effects of somatic variants by PolyPhen and SIFT were finally selected to be damaging and/or deleterious in our study.

## MATERIALS AND METHODS

### Copy number analysis

We have previously performed copy number analysis for the 8 legacy Wilms tumor samples reported by Cowell and Hawthorn [10]. The TARGET samples were analyzed using Partek Genomics Suite 6.6 (Partek Inc., St Louis, MO, USA) for LOH and copy number alterations (CNAs) using paired .CEL and .CHP files, which were generated using Genome-Wide Human 6.0 SNP arrays (Affymetrix), from the TARGET database. For CNA analysis, the genomic segmentation algorithm available in Partek Genomics Suite was employed using default parameters, including minimum genomics markers: 50, signal to noise ratio: 0.3, segmentation p-value: 0.01. LOH was analyzed using the Hidden Markov model (HMM) algorithm with default parameters, max probability: 0.9999 and genotype error: 0.01.

### DNA samples for exome sequencing

Genomic DNAs were prepared from 8 WT tissues (GOS100, 11, 132, 32, 52, 54, 576, 900), using standard phenol/chloroform extraction procedures or Wizard Genomics DNA purification kits (Madison, WI). Tumor samples were collected immediately following surgical resection during the period 1982–1992 and snap frozen in liquid nitrogen. At the time of collection, the diagnosis of WT was confirmed histopathologically, although in some cases the specific stage was not recorded. Unfortunately, it has also not been possible to recover this information retrospectively from some of the anonymized legacy samples. DNA was prepared from the whole tumor sample from snap frozen tissue using standard phenol/chloroform extraction procedures. Supplemental TableS1 shows the clinical information available for each sample used in the study and specifies which analyses were performed on the individual samples. 

### Exome sequencing

Exon capture libraries were constructed from 1 ug of genomic DNA using the Agilent Human All Exon Target Enrichment kit (38 Mb) or the SureSelect Human All Exon 50 Mb kit according to the manufacturer’s protocol (Agilent, Santa Clara, CA). The individual libraries were quality-checked and quantified using the Agilent 2100 Bioanalyzer and SYBR Green-qPCR (BIO-RAD, Hercules, CA), respectively. The libraries were sequenced on the Illumina HiSeq 2500 ver.2 (Illumina, San Diego, CA) using paired-end, 50 bp cycles. Base calling, reads quality assessment, de-multiplexing, and transferring to a fastq format was performed using Illumina data analysis software. FastQC was used for Quality control of the sequence reads. The average number of reads was 50 million/sample and on average, 92 % of total reads uniquely mapped on the NCBI37/hg19 reference sequence. 

### Exome data analysis

Reads that passed quality control were then aligned to the human reference genome (hg19) using BWA (Burrow-Wheelers Aligner version 0.6.1) with default settings. The generated BAM files were imported into Genome Analysis Toolkit, GATK2, for removal of duplicates, local realignment, mate-pair fixation, re-calibration and then variants were detected by HaplotypeCaller with a cutoff of depth at least 5. Germline variants were excluded by subtracting normal genotypes from tumor profiles using bedtools. In the case of our 8 legacy samples, we had lymphblastoid cell lines from 3 patients, for the remaining 5 patients we used the pooled lymphoblastoid cell line data as a baseline. The somatic variants resulting from these analyses were annotated using Ensemble variant effect predictor, VEP (release ver. 75). In the same way, 42 matched tumor-normal WTs, available through the TARGET study, were analyzed to search for somatic variants on chr16q. These annotated data were then filtered to investigate only those variants that were predicted to cause protein dysfunction and included deletions and insertions that lead to frame shifts, variants in critical splice junction nucleotides and missense variants generating stop codons or leading to predicted deleterious events in the protein. SIFT (Sort Intolerant From Tolerant) [11] and/ or PolyPhen-2 (Polymorphism Phenotyping v.2.) [12] were used to annotate the damaging and deleterious effects of missense variants.

For analysis of 16q specific somatic alterations, the data were filtered to include only altered alleles on chr16q. The annotated variants from exome data were categorized according to the chr16q structural abnormalities as determined by microarray analysis resulting in 25 WTs with16qdel/LOH and 22 WTs with wild-type chr16q profiles (16qN). For 16qdel/LOH samples, variants caused by deletion were sorted according to the ratio of read depths between a reference and an altered allele for each position and filtered to include variants with >=80% of altered allele ratio in tumor samples and <= 75% in the matched control samples with consideration for normal tissue contamination. 

Several pathway analysis programs were used to analyze the data, including Ingenuity Pathway Analysis (IPA, http://www.qiagenbioinformatics.com ), Database for Annotation, Visualization and Integrated Discovery (DAVID v6.8. http://software.broadinstitute.org/gsea/msigdb/annotate.jsp ), Geneset Enrichment Analysis (GSEA, https://david.ncifcrf.gov/home.jsp ). Analysis of cilia-related gene overlaps was conducted using CilDB (http://cildb.cgm.cnrs-gif.fr ). For DAVID, a Fisher Exact P-Value is used to determine whether the proportion of genes falling into each category differs by group. The Fisher Exact is adopted to measure gene enrichment in annotation terms. We have reported Enrichment scores for each of the Functional Annotation clusters which is based on the p-values for each of the term members. An FDR q-value=0.05 was used to compute overlaps in GSEA Hallmark Genesets.

## CONCLUSION

In summary, we have found that 49% of WT samples carry a del/LOH event on chromosome 16q when the patient cohort is preselected as high risk. These findings support a number of other studies implicating structural abnormalities of 16q with more aggressive form of WT. Studies aimed at identifying causative genes in minimal regions of overlap in patients with del/LOH at 16q have been largely unsuccessful and this may be due to multiple gene variants mapping to that region of the genome. We have identified a series of WT samples with chromosome16q del/LOH and defined deleterious variants in genes mapping to that region. We propose that increased ciliary dysfunction may be responsible of the more aggressive nature of WT with chromosome 16q del/LOH. 

## SUPPLEMENTARY MATERIALS AND FIGURES



## SUPPLEMENTARY MATERIALS AND TABLES



## Abbreviations

**Acronymx**: **Description**
16Nx: tumors with no loss of 16q
16qdel/LOHx: Tumors with deletion or LOH of 16q
ABCC12x: ATP-binding cassette, sub-family C (CFTR/MRP), member 12
ACANx: aggrecan
ACSF3x: acyl-CoA synthetase family member 3
ADAMTS18x: DAM metallopeptidase with thrombospondin type 1 motif, 18
ADPKDx: Autosomal Dominant Polycystic Kidney Disease
ALDH1B1x: aldehyde dehydrogenase 1 family, member B1
ALPK2x: alpha-kinase 2
AMER1x: APC membrane recruitment protein 1
ANKRD36x: ankyrin repeat domain 36
APCx: Adenomatous polyposis coli
APOBx: apolipoprotein B
ARID1Ax: AT rich interactive domain 1A
ASXL1x: additional sex combs like 1
ATMx: ataxia telangiectasia mutated
AXINx: Axis Inhibition Protein
BCL-9x: B-Cell Lymphoma-9
BCLAF1x: BCL2-associated transcription factor 1
BCMO1x: beta-carotene 15,15’-monooxygenase 1
BCORx: BCL6 Corepressor
BCORL1x: BCL6 Corepressor Like 1
BPIFB4x: BPI fold containing family B, member 4
BWAx: Burrow-Wheelers Aligner
c-JUNx: Jun Oncogene
CA6x: carbonic anhydrase VI
CABIN1x: calcineurin binding protein 1
CACNA1Bx: calcium channel, voltage-dependent, N type, alpha 1B subunit
CCDC135x: coiled-coil domain containing 135
CDH11x: cadherin 11
CDH3x: cadherin 3
CDKN2Ax: cyclin-dependent kinase inhibitor 2A
CES1x: carboxylesterase 1
CHST5x: carbohydrate (N-acetylglucosamine 6-O) sulfotransferase 5
CMYA5x: cardiomyopathy associated 5
CNAx: Copy Number Alteration
CNGB1x: cyclic nucleotide gated channel beta 1
COL4A3x: collagen, type IV, alpha 3
COL6A3x: Collagen Type VI Alpha 3 Chain
CTNNB1x: catenin (cadherin-associated protein), beta 1
CTU2x: cytosolic thiouridylase subunit 2 homolog
DAVIDx: Database for Annotation, Visualization and Integrated Discovery
DAWTx: Diffuse Anaplastic Wilms Tumor
delx: deletion
DGCR8x: DGCR8 microprocessor complex subunit
DICER1 x: dicer 1, ribonuclease type III
DNAx: Deoxyribonucleic Acid
DNAH11x: dynein, axonemal, heavy chain 11
DNAH5x: dynein, axonemal, heavy chain 5
DRC7x: dyenin regulatory complex 7
DROSHA x: drosha, ribonuclease type III
ENKD1x: enkurin domain containing 1
FCGBPx: Fc fragment of IgG binding protein
FDZ9x: Fizzled 9
FHWTx: Favorable Histology Wilms Tumor
GAS8x: growth arrest-specific 8
GATKx: Genome Analysis Toolkit
GLI3x: GLI family zinc finger 3
GLIS1x: GLIS family zinc finger 1
GPR98x: G protein-coupled receptor 98
GPRIN2x: G protein regulated inducer of neurite outgrowth 2
GSEAx: Geneset Enrichment Analysis
HMMx: Hidden Markov Model
HSD17B2x: hydroxysteroid (17-beta) dehydrogenase 2
HYDINx: axonemal central pair apparatus protein
IPAx: Ingenuity Pathway Analysis
KCNG4x: potassium voltage-gated channel, subfamily G, member 4
KCNJ12x: potassium inwardly-rectifying channel, subfamily J, member 12
KREMENx: Kringle domain-containing transmembrane protein
KRT32x: keratin 32
KRT32x: keratin 32
KRT37x: keratin 37
LOHx: Loss of Heterozygosity
MAP2K3x: mitogen-activated protein kinase kinase 3
MAP3K4x: Mitogen-Activated Protein Kinase Kinase Kinase 4
MAXx: MYC Associated Factor X
MC1Rx: melanocortin 1 receptor
MDM2x: Mouse Double Minute 2
MKI67x: marker of proliferation Ki-67
MKSx: Merkel Syndrome
MLLT1x: mixed-lineage leukemia (trithorax homolog, Drosophila); translocated to, 1
MT1Ax: metallothionein 1A
mTORx: mammalian Target of Rapamycin
MUC20x: mucin 20, cell surface associated
MUC3Ax: mucin 3A
MYCNx: v-myc avian myelocytomatosis viral oncogene neuroblastoma derived homolog
NEBx: nebulin
NFATx: nuclear factor of activated T-cells
NONOx: Non-POU Domain Containing Octamer Binding
NPHPx: Nephronophthisis
NPIPB15x: nuclear pore complex interacting protein family, member B15
NQO1x: NAD(P)H dehydrogenase, quinone 1
NRAPx: nebulin-related anchoring protein
NRAPx: nebulin-related anchoring protei
NWTSGx: National Wilms’ Tumor Study Group
OBSCNx: obscurin,
OR2B11x: olfactory receptor, family 2, subfamily B, member 11
OR4C3x: olfactory receptor, family 4, subfamily C, member 3
OR9G1x: olfactory receptor, family 9, subfamily G, member 1
PCDx: Primary Ciliary Dyskinesia
PCNTx: pericentrin
PDE4DIPx: phosphodiesterase 4D interacting protein
PDGFRx: Platelet-derived growth factor
PHLPP2x: PH domain and leucine rich repeat protein phosphatase 2
PIK3R1x: phosphoinositide-3-kinase, regulatory subunit 1 (alpha)
PKDx: Polycystic Kidney Disease
PKD1x: polycystic kidney disease 1
PKD1L2x: polycystic kidney disease 1-like 2
PKD2x: polycystic kidney disease 2
PLDx: phospholipase D family,
PLIN4x: perilipin 4
PMFBP1x: polyamine modulated factor 1 binding protein 1
POLYPHENx: Polymorphism Phenotyping
PPP3CAx: protein phosphatase 3, catalytic subunit, alpha isozyme
PRIM2x: primase, DNA, polypeptide 2
PRKAG2 x: protein kinase, AMP-activated, gamma 2 non-catalytic subunit
PTCH1x: Patched 1
PTCHD3x: patched domain containing 3
PTPRHx: protein tyrosine phosphatase, receptor type, H
PZPx: pregnancy-zone protein
RHBGx: Rh family, B glycoprotein
RNF43x: ring finger protein 43
RP1L1x: retinitis pigmentosa 1-like 1
RPGRIP1Lx: retinitis pigmentosa GTPase regulator interacting protein 1 like
SCGB1C1x: secretoglobin, family 1C, member 1
SHhx: Sonic Hedgehog
SIFTx: Sort Intolerant From Tolerant
SIX1x: SIX homeobox 1
SIX2x: SIX homeobox 2
SMOx: smoothened, frizzled class receptor
SNPx: Single Nucleotide Polymorphism
SOX7x: SRY (sex determining region Y)-box 7
STK3x: serine/threonine kinase 3
TAF1Cx: TATA box binding protein (TBP)-associated factor
TARGETx: Therapeutically Applicable Research to Generate Effective Treatments
TGF-B x: Transforming Growth Factor-Beta
TMEM231x: transmembrane protein 231
TP53x: tumor protein p53
TRPC1x: transient receptor potential cation channel, subfamily C, member 1
TRPV4 x: transient receptor potential cation channel, subfamily V, member 4
TUBB3x: tubulin, beta 3 class III
TZx: Transition Zone
UKCCSGx: United Kingdom Children’s Cancer Study Group
VEP x: Variant Effects Predictor
Wntx: Wingless/Integrated
WNT11x: wingless-type MMTV integration site family, member 11
WTx: Wilms Tumor
WT1x: Wilms tumor 1
XPO5x: exportin 5
ZCCHC14x: zinc finger, CCHC domain containing 14
ZNF19x: zinc finger protein 19
ZNF778x: zinc finger protein 778

## References

[1] Deng C, Dai R, Li X, Liu F (2016). Genetic variation frequencies in Wilms’ tumor: A meta-analysis and systematic review.. Cancer Sci.

[2] Brown E, Hebra A, Jenrette J, Hudspeth M (2010). Successful treatment of late, recurrent wilms tumor with high-dose chemotherapy and autologous stem cell rescue in third complete response.. J Pediatr Hematol Oncol.

[3] Dome JS, Perlman EJ, Graf N (2014). Risk stratification for wilms tumor: current approach and future directions.. Am Soc Clin Oncol Educ Book.

[4] Breslow NE, Olson J, Moksness J, Beckwith JB, Grundy P (1996). Familial Wilms’ tumor: a descriptive study.. Med Pediatr Oncol.

[5] Grundy PE, Breslow NE, Li S, Perlman E, Beckwith JB, Ritchey ML, Shamberger RC, Haase GM, D’Angio GJ, Donaldson M, Coppes MJ, Malogolowkin M, Shearer P, National Wilms Tumor Study Group (2005). Loss of heterozygosity for chromosomes 1p and 16q is an adverse prognostic factor in favorable-histology Wilms tumor: a report from the National Wilms Tumor Study Group.. J Clin Oncol.

[6] Messahel B, Williams R, Ridolfi A, A’hern R, Warren W, Tinworth L, Hobson R, Al-Saadi R, Whyman G, Brundler MA, Kelsey A, Sebire N, Jones C, Children’s Cancer and Leukaemia Group (CCLG) (2009). Allele loss at 16q defines poorer prognosis Wilms tumour irrespective of treatment approach in the UKW1-3 clinical trials: a Children’s Cancer and Leukaemia Group (CCLG) Study.. Eur J Cancer.

[7] Grundy PE, Telzerow PE, Breslow N, Moksness J, Huff V, Paterson MC (1994). Loss of heterozygosity for chromosomes 16q and 1p in Wilms’ tumors predicts an adverse outcome.. Cancer Res.

[8] Pan Z, He H, Tang L, Bu Q, Cheng H, Wang A, Lyu J, You H (2017). Loss of heterozygosity on chromosome 16q increases relapse risk in Wilms’ tumor: a meta-analysis.. Oncotarget.

[9] Knudson AG (2001). Two genetic hits (more or less) to cancer.. Nat Rev Cancer.

[10] Hawthorn L, Cowell JK (2011). Analysis of wilms tumors using SNP mapping array-based comparative genomic hybridization.. PLoS One.

[11] Ng PC, Henikoff S (2001). Predicting deleterious amino acid substitutions.. Genome Res.

[12] Ramensky V, Bork P, Sunyaev S (2002). Human non-synonymous SNPs: server and survey.. Nucleic Acids Res.

[13] Gadd S, Huff V, Walz AL, Ooms AH, Armstrong AE, Gerhard DS, Smith MA, Auvil JM, Meerzaman D, Chen QR, Hsu CH, Yan C, Nguyen C (2017). A Children’s Oncology Group and TARGET initiative exploring the genetic landscape of Wilms tumor.. Nat Genet.

[14] Skotnicka-Klonowicz G, Rieske P, Bartkowiak J, Szymik-Kantorowicz S, Daszkiewicz P, Debiec-Rychter M (2000). 16q heterozygosity loss in Wilms’ tumour in children and its clinical importance.. Eur J Surg Oncol.

[15] Wittmann S, Zirn B, Alkassar M, Ambros P, Graf N, Gessler M (2007). Loss of 11q and 16q in Wilms tumors is associated with anaplasia, tumor recurrence, and poor prognosis.. Genes Chromosomes Cancer.

[16] Fukuzawa R, Anaka MR, Morison IM, Reeve AE (2017). The developmental programme for genesis of the entire kidney is recapitulated in Wilms tumour.. PLoS One.

[17] Marra AN, Li Y, Wingert RA (2016). Antennas of organ morphogenesis: the roles of cilia in vertebrate kidney development.. Genesis.

[18] Ito J, Johnson WW (1969). Ultrastructure of Wilms’ tumor. I. Epithelial cell.. J Natl Cancer Inst.

[19] Katz SM, Morgan JJ (1984). Cilia in the human kidney.. Ultrastruct Pathol.

[20] Duffy JL, Suzuki Y (1968). Ciliated human renal proximal tubular cells. Observations in three cases of hypercalcemia.. Am J Pathol.

[21] Ong AC, Wagner B (2005). Detection of proximal tubular motile cilia in a patient with renal sarcoidosis associated with hypercalcemia.. Am J Kidney Dis.

[22] Boerwinkle C, Marshall JD, Bryant J, Gahl WA, Olivier KN, Gunay-Aygun M (2017). Respiratory manifestations in 38 patients with Alström syndrome.. Pediatr Pulmonol.

[23] Yang Y, Cochran DA, Gargano MD, King I, Samhat NK, Burger BP, Sabourin KR, Hou Y, Awata J, Parry DA, Marshall WF, Witman GB, Lu X (2011). Regulation of flagellar motility by the conserved flagellar protein CG34110/Ccdc135/FAP50.. Mol Biol Cell.

[24] Raidt J, Wallmeier J, Hjeij R, Onnebrink JG, Pennekamp P, Loges NT, Olbrich H, Häffner K, Dougherty GW, Omran H, Werner C (2014). Ciliary beat pattern and frequency in genetic variants of primary ciliary dyskinesia.. Eur Respir J.

[25] Geremek M, Ziętkiewicz E, Bruinenberg M, Franke L, Pogorzelski A, Wijmenga C, Witt M (2014). Ciliary genes are down-regulated in bronchial tissue of primary ciliary dyskinesia patients.. PLoS One.

[26] Kim TW, Kim YJ, Kim HT, Park SR, Lee MY, Park YD, Lee CH, Jung JY (2016). NQO1 Deficiency Leads Enhanced Autophagy in Cisplatin-Induced Acute Kidney Injury Through the AMPK/TSC2/mTOR Signaling Pathway.. Antioxid Redox Signal.

[27] Olbrich H, Cremers C, Loges NT, Werner C, Nielsen KG, Marthin JK, Philipsen M, Wallmeier J, Pennekamp P, Menchen T, Edelbusch C, Dougherty GW, Schwartz O (2015). Loss-of-Function GAS8 Mutations Cause Primary Ciliary Dyskinesia and Disrupt the Nexin-Dynein Regulatory Complex.. Am J Hum Genet.

[28] Jeanson L, Thomas L, Copin B, Coste A, Sermet-Gaudelus I, Dastot-Le Moal F, Duquesnoy P, Montantin G, Collot N, Tissier S, Papon JF, Clement A, Louis B (2016). Mutations in GAS8, a Gene Encoding a Nexin-Dynein Regulatory Complex Subunit, Cause Primary Ciliary Dyskinesia with Axonemal Disorganization.. Hum Mutat.

[29] Lewis WR, Malarkey EB, Tritschler D, Bower R, Pasek RC, Porath JD, Birket SE, Saunier S, Antignac C, Knowles MR, Leigh MW, Zariwala MA, Challa AK (2016). Mutation of Growth Arrest Specific 8 Reveals a Role in Motile Cilia Function and Human Disease.. PLoS Genet.

[30] Evron T, Philipp M, Lu J, Meloni AR, Burkhalter M, Chen W, Caron MG (2011). Growth Arrest Specific 8 (Gas8) and G protein-coupled receptor kinase 2 (GRK2) cooperate in the control of Smoothened signaling.. J Biol Chem.

[31] Shi X, Garcia G, Van De Weghe JC, McGorty R, Pazour GJ, Doherty D, Huang B, Reiter JF (2017). Erratum: super-resolution microscopy reveals that disruption of ciliary transition-zone architecture causes Joubert syndrome.. Nat Cell Biol.

[32] Wang L, Dynlacht BD (2018). The regulation of cilium assembly and disassembly in development and disease.. Development.

[33] Khan NA, Willemarck N, Talebi A, Marchand A, Binda MM, Dehairs J, Rueda-Rincon N, Daniels VW, Bagadi M, Thimiri Govinda Raj DB, Vanderhoydonc F, Munck S, Chaltin P, Swinnen JV (2016). Identification of drugs that restore primary cilium expression in cancer cells.. Oncotarget.

[34] Vertii A, Bright A, Delaval B, Hehnly H, Doxsey S (2015). New frontiers: discovering cilia-independent functions of cilia proteins.. EMBO Rep.

[35] Pala R, Alomari N, Nauli SM (2017). Primary Cilium-Dependent Signaling Mechanisms.. Int J Mol Sci.

[36] Pressey JG, Anderson JR, Crossman DK, Lynch JC, Barr FG (2011). Hedgehog pathway activity in pediatric embryonal rhabdomyosarcoma and undifferentiated sarcoma: a report from the Children’s Oncology Group.. Pediatr Blood Cancer.

[37] Oue T, Uehara S, Yamanaka H, Takama Y, Oji Y, Fukuzawa M (2011). Expression of Wilms tumor 1 gene in a variety of pediatric tumors.. J Pediatr Surg.

[38] Wang Y, Zhou CJ, Liu Y (2018). Wnt Signaling in Kidney Development and Disease.. Prog Mol Biol Transl Sci.

[39] Chung E, Deacon P, Marable S, Shin J, Park JS (2016). Notch signaling promotes nephrogenesis by downregulating Six2.. Development.

[40] Chung E, Deacon P, Park JS (2017). Notch is required for the formation of all nephron segments and primes nephron progenitors for differentiation.. Development.

[41] Li H, Rao A, Hogan PG (2004). Structural delineation of the calcineurin-NFAT interaction and its parallels to PP1 targeting interactions.. J Mol Biol.

[42] Nguyen AH, Béland M, Gaitan Y, Bouchard M (2009). Calcineurin a-binding protein, a novel modulator of the calcineurin-nuclear factor of activated T-cell signaling pathway, is overexpressed in wilms’ tumors and promotes cell migration.. Mol Cancer Res.

[43] Lemos FO, Ehrlich BE (2018). Polycystin and calcium signaling in cell death and survival.. Cell Calcium.

[44] Ravichandran K, Edelstein CL (2014). Polycystic kidney disease: a case of suppressed autophagy?. Semin Nephrol.

[45] Lv W, Booz GW, Wang Y, Fan F, Roman RJ (2018). Inflammation and renal fibrosis: recent developments on key signaling molecules as potential therapeutic targets.. Eur J Pharmacol.

