# Supplementary material for: Variant profiles of genes mapping to chromosome 16q loss in Wilms tumors reveals link to cilia-related genes and pathways
Source: Genes Cancer. 2020 Oct 6;11(3-4):137–53. doi: 10.18632/genesandcancer.207 (PMC7805536; doi:10.18632/genesandcancer.207)
Supplement: Supplementary file 1 [file ganc-11-137-s001.pdf]

Variant profiles of genes mapping to chromosome 16q loss in Wilms tumors reveals link to cilia-related genes and pathways – Kitamura et al

Supplementary Images 1: Established Cilia-related Pathways. Genes with detected deleterious and/or damaging mutations are shown for 16qloss/LOH samples in red and 16qN samples outlined in blue.

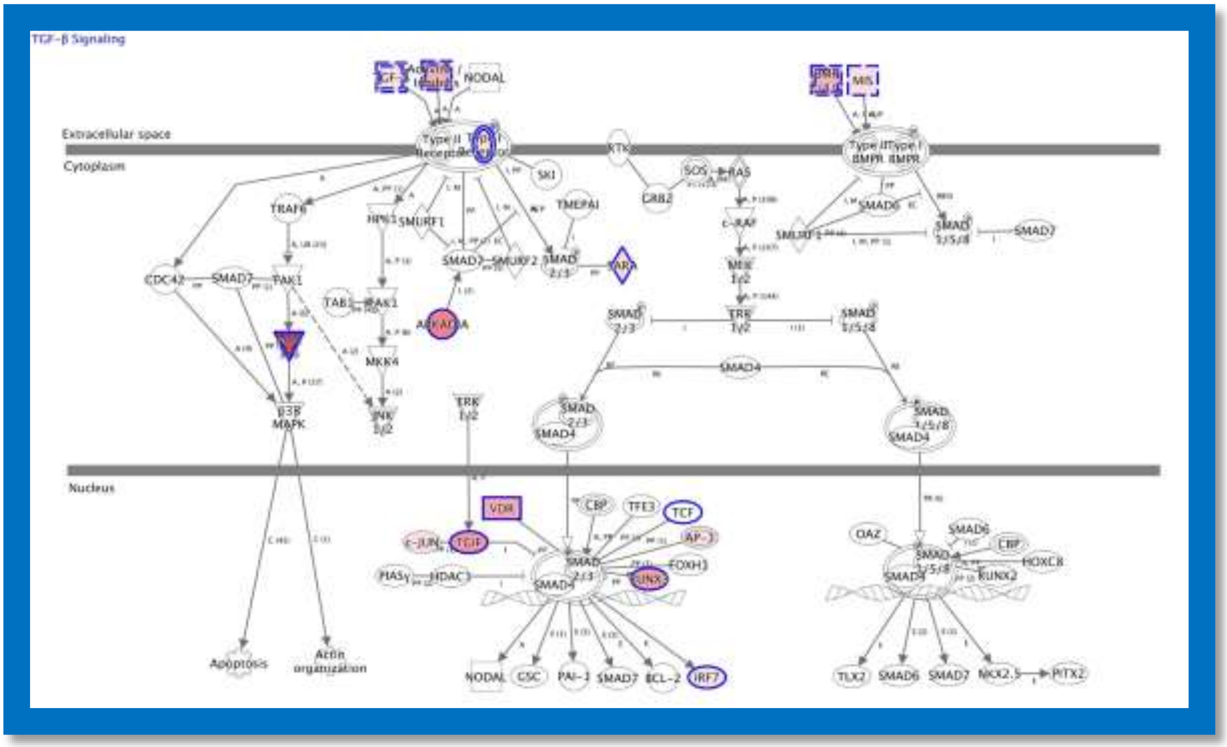

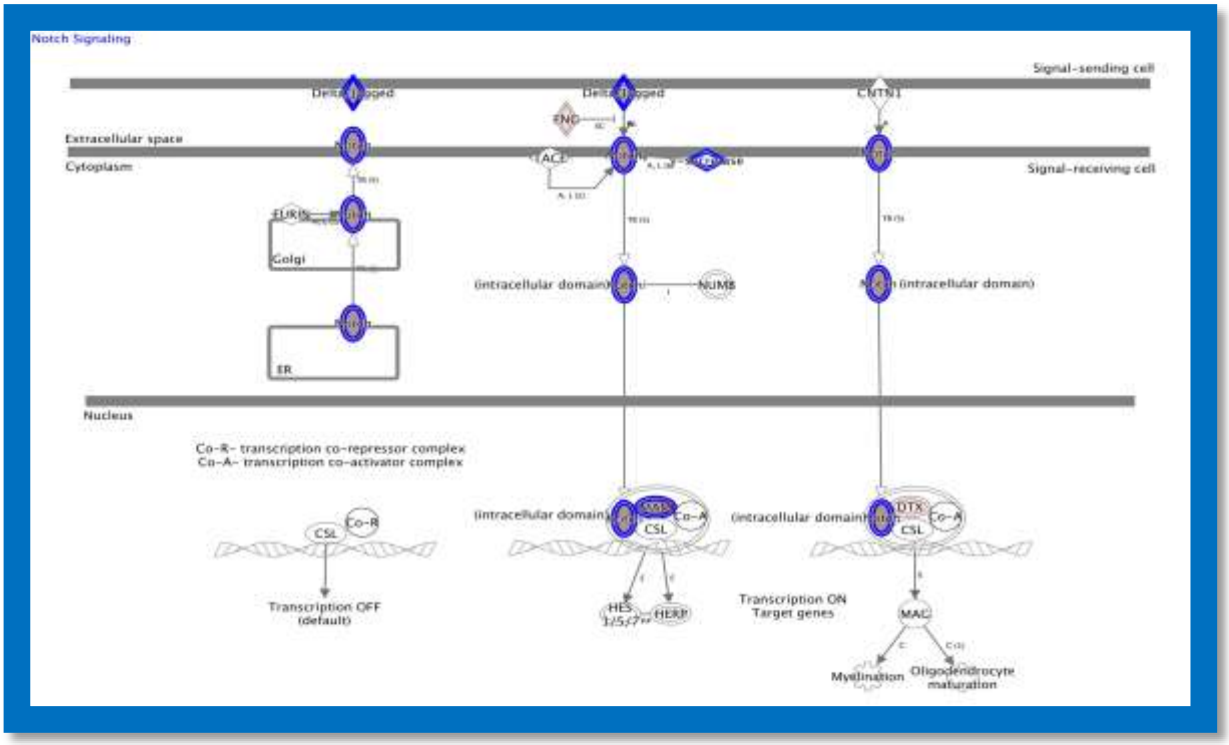

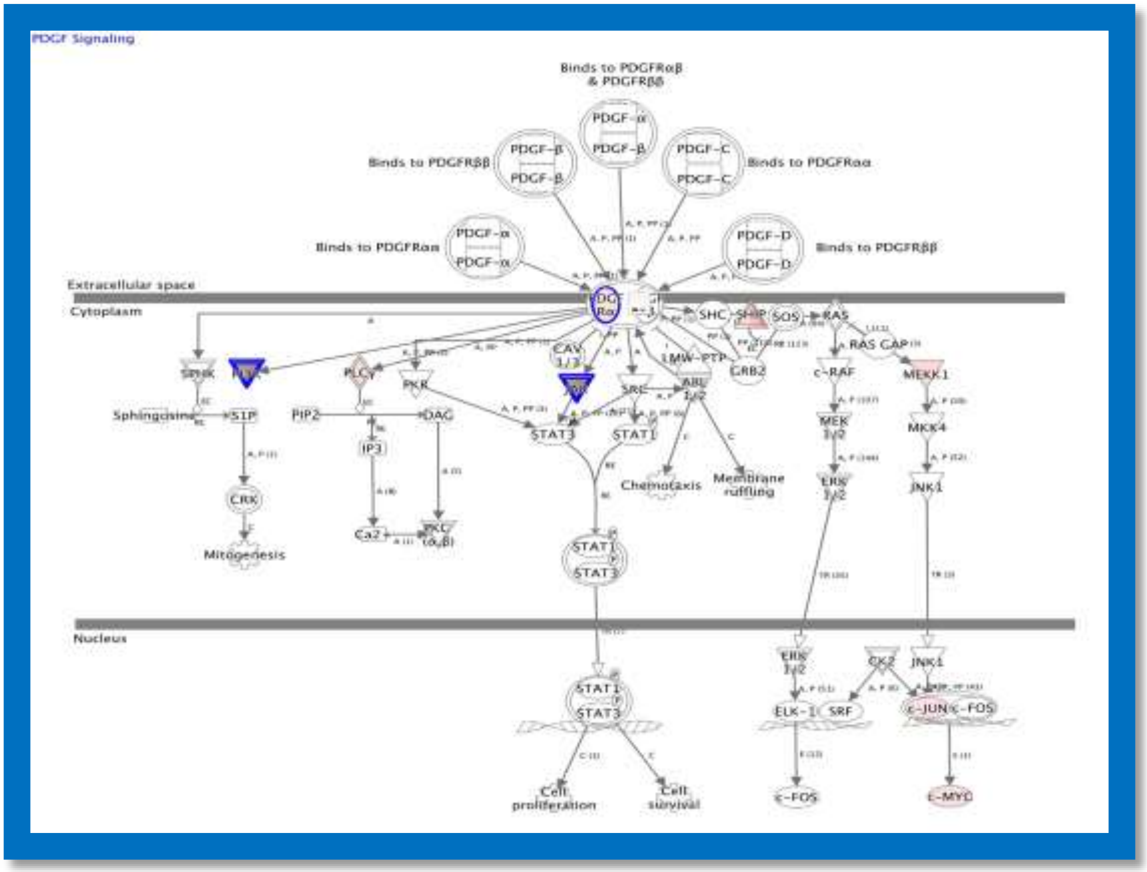
PDGF Signaling
Binds to PDGFRαβ & PDGFRββ
PDGF-α
PDGF-β
Binds to PDGFRββ
PDGF-C
Binds to PDGFRαα
PDGF-D
Extracellular space
Cytoplasm
SHC
SHIP
SOS
RAS
RAS GAP
CAV 1/2
LMW-PTP
GRB2
c-RAF
MEKK1
SPHK
PLCγ
PKR
SRC
ABL 1/2
Sphingosine
S1P
PIP2
DAG
IP3
STAT3
STAT1
MEK 1/2
MKK4
Chemotaxis
Membrane ruffling
ERK 1/2
JNK1
CRK
Mitogenesis
Ca2+
PKC (α,β)
Nucleus
CK2
ELK-1
SRF
c-JUN
c-FOS
Cell proliferation
Cell survival
c-MYC

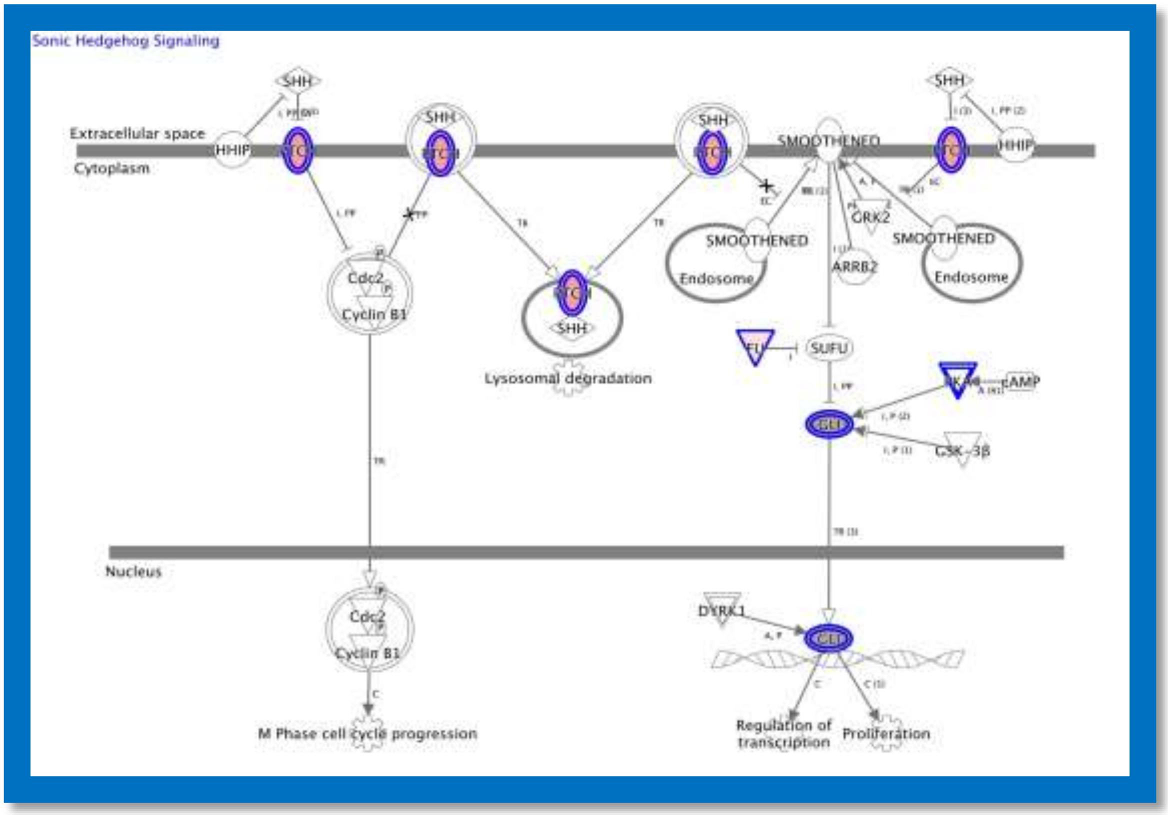
Sonic Hedgehog Signaling
SHH
Extracellular space
Cytoplasm
HHIP
SMOOTHENED
GRK2
Cdc2
Cyclin B1
Endosome
ARRB2
Lysosomal degradation
SUFU
cAMP
GSK-3β
Nucleus
DYRK1
M Phase cell cycle progression
Regulation of transcription
Proliferation

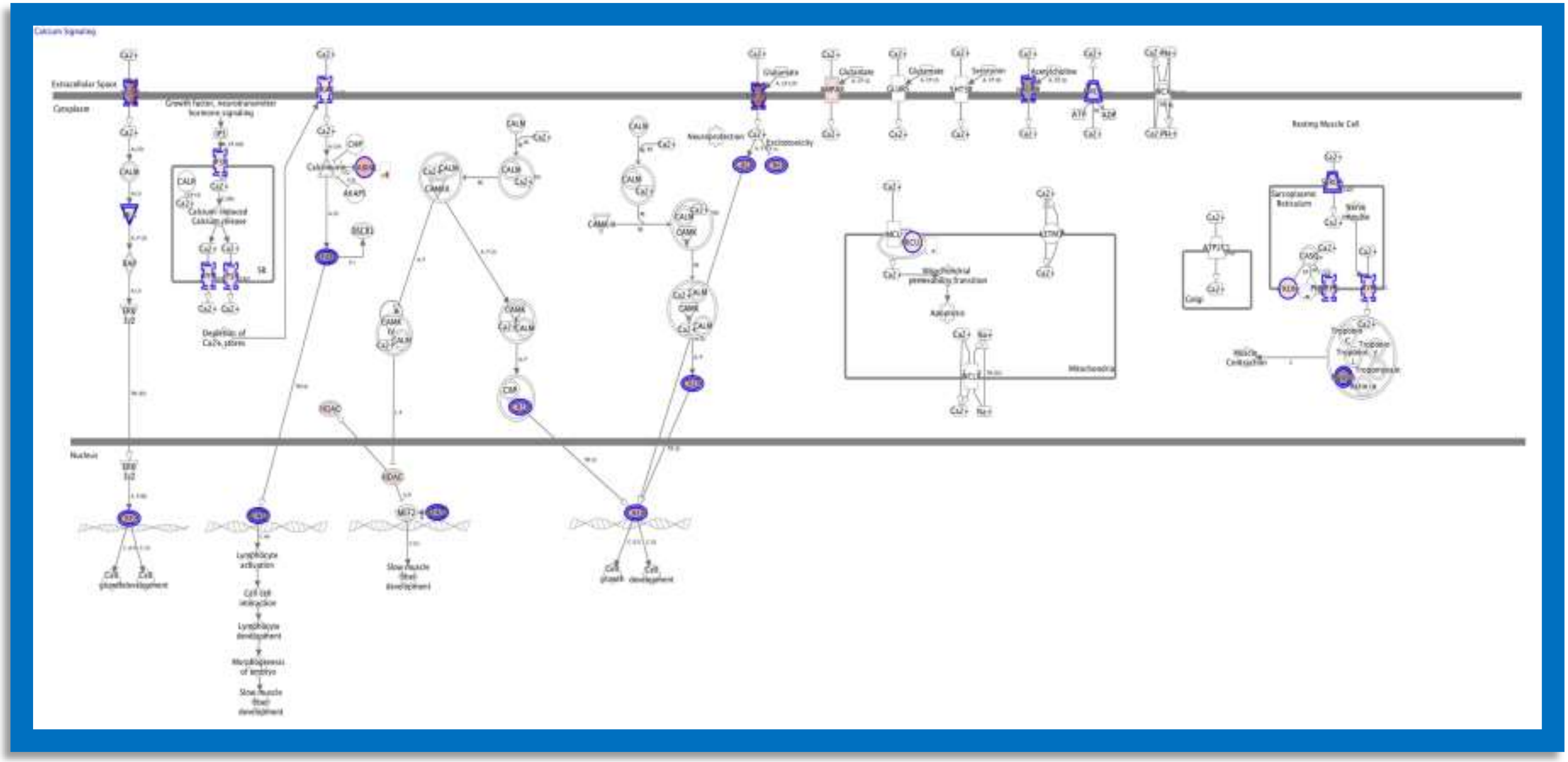

Calcium Signaling
Extracellular Space
Cytoplasm
Nucleus
Growth factors, neurotransmitter hormone signaling
Depletion of Ca2+ stores
Neuroprotection
Excitotoxicity
Glutamate
Serotonin
Acetylcholine
Mitochondria
Mitochondrial permeability transition
Apoptosis
Resting Muscle Cell
Sarcoplasmic Reticulum
Golgi
Muscle Contraction
Tropomyosin
Lymphocyte activation
Cell-cell interaction
Lymphocyte development
Morphogenesis of embryo
Slow muscle fiber development
Cell growth
Cell development
